# Brassinosteroids Attenuate Moderate High Temperature-Caused Decline in Tea Quality by Enhancing Theanine Biosynthesis in *Camellia sinensis* L.

**DOI:** 10.3389/fpls.2018.01016

**Published:** 2018-07-24

**Authors:** Xin Li, Ji-Peng Wei, Golam J. Ahammed, Lan Zhang, Yang Li, Peng Yan, Li-Ping Zhang, Wen-Yan Han

**Affiliations:** ^1^Key Laboratory of Tea Quality and Safety Control, Ministry of Agriculture, Tea Research Institute, Chinese Academy of Agricultural Sciences, Hangzhou, China; ^2^Graduate School of Chinese Academy of Agricultural Sciences, Beijing, China; ^3^College of Forestry, Henan University of Science and Technology, Luoyang, China

**Keywords:** Brassinosteroids, *Camellia sinensis*, high temperature, summer tea, tea quality, theanine

## Abstract

Temperature is a major environmental signal that governs plant growth and development. A moderately high ambient temperature alters plant metabolism without significant induction of heat–stress responses. Despite ancillary reports on the negative effect of warmer climate on tea quality, information on specific effect of sub high temperature (SHT) on theanine accumulation is scanty. L-Theanine is the most abundant free amino acid in tea (*Camellia sinensis* L.) leaves that contributes to the unique umami flavor of green tea infusion. Tea harvested in warmer months lacks distinctive umami taste due to low theanine content. In this study, we showed that SHT (35°C) gradually decreased theanine concentration over time, which was closely associated with the SHT-induced suppression in theanine biosynthetic genes. 24-epibrassinolide (BR), a bioactive brassinosteroids, attenuated the SHT-induced reduction in theanine concentration by upregulating the transcript levels of theanine biosynthetic genes, such as *ARGININE DECARBOXYLASE* (*CsADC*), *GLUTAMINE SYNTHETASE* (*CsGS*), *GLUTAMATE SYNTHASE* (*CsGOGAT*) and *THEANINE SYNTHASE* (*CsTS*). Furthermore, time-course analysis of the activity of theanine biosynthetic enzyme reveals that BR-induced regulation of GS and GOGAT activity plays essential role in maintaining theanine content in tea leaves under SHT, which is consistent with the central position of GOGAT in theanine biosynthetic pathway. Therefore, it is convincing to propose that exogenous BR treatment can be advocated to improve summer tea quality by enhancing *in vivo* accumulation of theanine. However, a future challenge is to use this information on the role of BR in theanine biosynthesis and thermotolerance to further understand how BR may be tuned to benefit plant fitness for enhancing tea quality.

## Introduction

Tea is the second most popular beverage after water in the world (Tounekti et al., 2013). The popularity of tea is simultaneously attributed to its pleasant taste and medicinal values (Mancini et al., 2017). The versatile health benefits of optimal tea consumption are well connected to the composition of some phytochemicals in tea (Kim et al., 2009; Zhang and Tsao, 2016). These constituents are broadly classified into tea polyphenols and amino acids (Tounekti et al., 2013). In addition, the tea quality also depends on the composition of these individual constituents. For instance, theanine, which is a unique non-protein amino acid, is responsible for the so called ‘umami’ taste of tea (Vuong et al., 2011; Cheng et al., 2017). A good number of studies suggest that L-theanine plays a vital role in the health promoting effect of tea (Kim et al., 2009; Siamwala et al., 2013). Theanine has positive effect on cognition and neuronal health since it improves memory and learning ability by activating relative central neurotransmitters (Kim et al., 2009). In addition, theanine can reduce blood-pressure, mental stress and anxiety, and promote relaxation and concentration by inhibiting the negative effects of caffeine (Unno et al., 2017).

**FIGURE 1:**
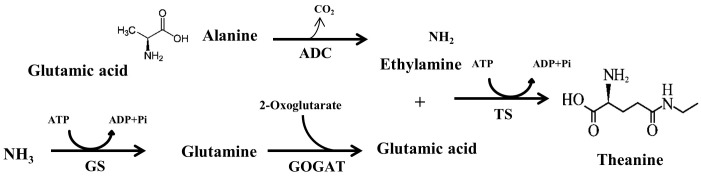
Theanine biosynthetic pathways in tea plants from NH_3_, glutamic acid, alanine and ethylamine. ADC, arginine decarboxylase; GS, glutamine synthetase; GOGAT, glutamate synthase; TS, theanine synthase. Adopted from Li et al. (2017b).

All theanine that naturally exist in tea plant belongs to L-theanine (Mu et al., 2015). According to the International Union of Pure and Applied Chemistry (IUPAC), L-theanine is named as 2-amino-4- (ethylcarbamoyl) butyric acid. It is also known as γ-glutamylethylamide that constitutes 50% of free amino acids in tea leaves (Vuong et al., 2011). L-theanine can be detected in almost all tissues in tea plants, however, major site of *in planta* synthesis is root, from where theanine is transported to the shoot (Deng et al., 2008). Theanine content is high in young leaves and declines with leaf maturity (Liu et al., 2017b). There are seventeen genes encoding enzymes involved in theanine metabolism, however, only several genes, including *THEANINE SYNTHASE* (*CsTS*), have positive correlation with theanine content in tea leaves (Liu et al., 2017b). In the theanine biosynthetic pathway, glutamic acid and ethylamine act as the immediate precursors of theanine, which are converted to L-theanine by the catalysis of TS (Cheng et al., 2017). In addition to TS, other enzymes responsible for the biosynthesis of L-theanine precursors, such as glutamine synthetase (GS), glutamate synthase (GOGAT) and alanine decarboxylase (ADC) play vital role in theanine accumulation in tea (**Figure 1**). In a recent study on effect of post-harvest spreading temperature on theanine level showed that 38°C temperature strongly suppressed the activity and gene expression levels of the theanine metabolism-related enzymes, leading to a reduction in theanine content (Liu et al., 2017a). This study highlights the significance of *CsGOGAT* in temperature-dependent spontaneous changes in theanine content during post-harvest processing of green tea.

Due to global warming, seasonal mean maximum temperatures increased remarkably in many regions of the world, which will further increase in the coming decades (Battisti and Naylor, 2009). Rising temperature, a key driving force of climate change, greatly affects plant growth and development. Recent studies show that climate change-related changes in global temperatures have profound effect on both yield and quality of tea (Ahmed et al., 2014). As plants are sessile, they have to face all these thermo-challenges in their natural ecosystems. While effect of extreme high temperatures on plant growth can be recognized by visible sign and yield reduction, sub high temperatures (SHT), which are just above physiological optimum, can cause massive metabolic changes without showing visible symptoms (Ahammed et al., 2016; Lu et al., 2017; Ohama et al., 2017). These responses are cumulative that comprise physiological, metabolic and transcriptional reprogramming (Liu Z.W. et al., 2016; Wu et al., 2016; Ohama et al., 2017). Particularly, in tea plants, high temperatures have specific effects on tea quality (Li et al., 2016). Seasonal studies show that tea grown in warmer climate, (e.g., summer tea) are bitter, which is well correlated to high polyphenol and low theanine contents (Xu et al., 2012). Spring tea (tea harvested in cool months) has the highest theanine content, which declines as the weather becomes warmer, indicating that high temperatures negatively impact theanine content in tea (Liu et al., 2017b). Due to more bitterness and astringency in summer and autumn teas, their economic values are low. For this reason, a large proportion of summer and autumn teas remains unharvested which results in a great waste of the total tea production (Dai et al., 2015). Therefore, it is indispensable to explore strategies that can potentially enhance theanine content in tea grown in warmer climate.

Brassinosteroids are a group of phytohormones that regulate multiple physiological and metabolic processes in plants (Ahammed et al., 2014; Li et al., 2016, 2017a). BRs also enhance tolerance to abiotic stress, including high temperature stress in a range of crop species (Ahammed et al., 2014, 2016). Previous studies reveal that stress ameliorative effects of BR are attributed to BR-induced enhancement in secondary metabolism in plants (Ahammed et al., 2012; Çoban and Göktürk Baydar, 2016; Li et al., 2016). Although a positive effect of BR on the biosynthesis of tea polyphenols and amino acids has been revealed, its effect on theanine content in tea leaves under high temperature conditions remains largely unclear. Despite considering the important roles of BR in theanine accumulation (Li et al., 2016), a few fundamental questions remain. For instance, how is the theanine accumulation affected in tea leaves under high temperature? How is the theanine biosynthesis regulated by BRs under high temperature in tea leaves? Therefore, we exposed tea plants to SHT with or without 24-epibrassinolide pretreatment to specifically explore their effects on theanine accumulation in tea leaves. The results show that theanine content gradually declines as the exposure duration progresses. However, exogenous BR can increase theanine concentration under SHT by regulating the activity of enzymes and genes involved in theanine biosynthesis. The results also suggest that regulation of theanine biosynthesis by BR can be exploited to improve both quality and medicinal value of summer tea.

## Materials and Methods

### Plant Materials, Growth Conditions and Treatments

Tea seedlings (*Camellia sinensis* L., cv. Longjing 43) were grown in a medium containing a mixture of peat, vermiculite and perlite (6:3:1, v:v:v) in plastic pots in controlled environment cabinets. One healthy seedling was grown per pot. The growth conditions were as follows: photosynthetic photon flux density (PPFD) of 600 μmol m^−2^ s^−1^, photoperiod of 12 h/12 h (day/night), day/night air temperature of 25/20°C and relative humidity of 80%. Seedlings were watered daily to maintain optimum moisture and were fertilized with Hoagland’s nutrient solution every 5 days. Two years-old tea seedlings were sprayed with 0.2 ppm 24-epibrassinolide (Sigma-Aldrich, St. Louis, MO, United States) as described previously (Li et al., 2016). Control seedlings were sprayed with double distilled water (ddH_2_O) containing the same ratio of organic solvent used to prepare working solution of BR. Twelve hours later, one half of the plants in each treatment were kept at 35°C temperature (SHT) for 5 days, whereas the other half (only BR) and the Control tea plants (CK) that did not receive BR pretreatment were grown under the normal conditions (25°C). Each treatment was replicated six times, and each replicate consisted of five seedlings. One bud and two leaves under the bud were sampled after 5 days (unless otherwise stated) of the SHT treatment for different biochemical analyses.

### Determination of Theanine Concentration

Theanine was extracted from the tea leaf samples with deionized water for 45 min in a water bath at 80°C, and the extract was filtered through a 0.45-μm Millipore filter before HPLC analysis. The theanine was then detected using an Shimadzu LC-20A HPLC system (Shimadzu, Japan) according to the method described by Tai et al. (2015).

### Assay of GS and GOGAT Activity

Total soluble protein from the tea leaf samples was extracted at 0°C temperature using a 200 mM PBS (pH 7.5) containing 5 mM EDTA, 12.5 mM 2-mercaptoethanol and 2 mM phenylmethyl sulphonyl fluoride. Coomassie Brilliant Blue was used to colorimetrically determine the protein content of the extract (Sedmark and Grossberg, 1977).

Glutamine synthetase (GS) activity assay was based on the replacement of the physiological substrate NH_4_^+^ by hydroxylamine. In principle, the formation of γ-glutamylhydroxamate (GHA) from hydroxylamine and glutamate is catalyzed by GS. GHA was colorimetrically analyzed at 540 nm after complexation with ferric chloride as described previously (Migge et al., 1997). In brief, the reaction mixture contained 100 μL imidazole buffer (450 mM, pH 7.2), 100 μL MgCl_2_ (450 mM), 100 μL NH_2_OH.HCl (60 mM), 100 μL ATP (80 mM), 100 μL L-glutamate (870 mM) and 300 μL H_2_O. The reaction was initiated by adding 50 μL protein extract. After incubation for 15 min at 30°C, the reaction was terminated by adding 850 μL of a solution containing 0.37 M FeCl_3_, 0.2 M trichloroacetic acid and 0.67 N HCl.

The extract for glutamine: 2-oxoglutarate aminotransferase (GOGAT) measurement was obtained by grinding 0.3 g frozen leaf sample in 2 ml 25 mM Tris–HCl buffer (pH 7.8) containing 1 mM MgCl_2_, 1 μM β-mercaptoethanol, 1 mM EDTA, and 1% (w/v) PVPP. For assays, the extract was centrifuged at 15,000 rpm for 20 min, and the GOGAT activity was measured in the supernatant as described by Suzuki et al. (2001).

### Total RNA Extraction and qPCR

Total RNA from tea leaf samples was isolated using TRIzol reagent (Invitrogen, Carlsbad, CA, United States) by the manufacturer’s instruction. Genomic DNA was removed from RNA samples with RNeasy Mini Kit (Qiagen, Germany). Reverse transcription was carried out by Superscript II (Invitrogen) following the manufacturer’s protocol. The primers used for qRT-PCR assay have been listed in Supplementary Table S1. qRT-PCR analysis was done on a Step One Plus Real-Time PCR system (Applied Biosystems, Foster City, CA, United States) with Power SYBR Green PCR Master Mix (Applied Biosystems). qRT-PCR conditions consisted of denaturation at 95°C for 3 min, followed by 40 cycles of denaturation at 95°C for 30 s, annealing at 58°C for 30 s and extension at 72 °C for 30 s. Transcript abundance was normalized to the polypyrimidine tract-binding protein (*PTB1*) gene as described previously (Li et al., 2018) and relative gene expression was calculated following formulae of Livak and Schmittgen (2001). Three biological replicates with three technical repeats were performed for each treatment.

### Statistical Analysis

Each biochemical assay had at least six replicates, and all data were expressed as the means ± standard deviations (SD). The data were statistically analyzed using SAS 8.1 software package (SAS Institute Inc., Cary, NC, United States), and the means were compared using Tukey’s test at the *P* < 0.05 level.

## Results

### Sub High Temperature Gradually Decreases Theanine Biosynthesis in Tea Leaves

**FIGURE 2:**
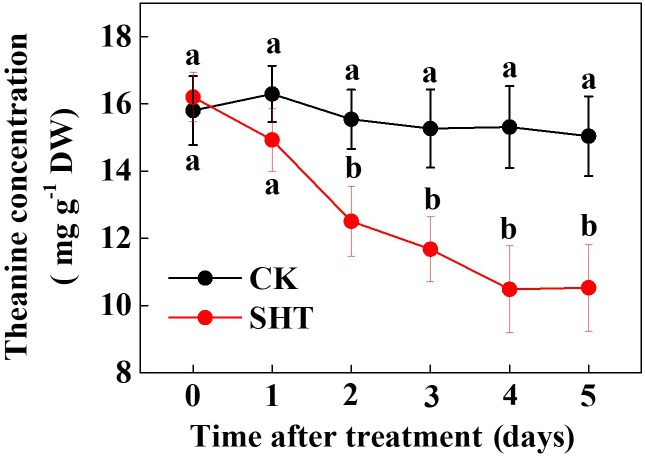
Time-course effect of sub high temperature (SHT) on theanine concentration in tea leaves. Two years-old tea [*C. sinensis* (L.) O. Kuntze cv. Longjing 43] seedlings were exposed to SHT (35°C) for 5 days in temperature-controlled chambers. One bud and two leaves under the bud were sampled at the indicated time-points to analyze theanine concentration. Data are presented as the mean of six replicates (±SD). Different letters indicate significant differences (*P* < 0.05) according to the Tukey’s test.

**FIGURE 3:**
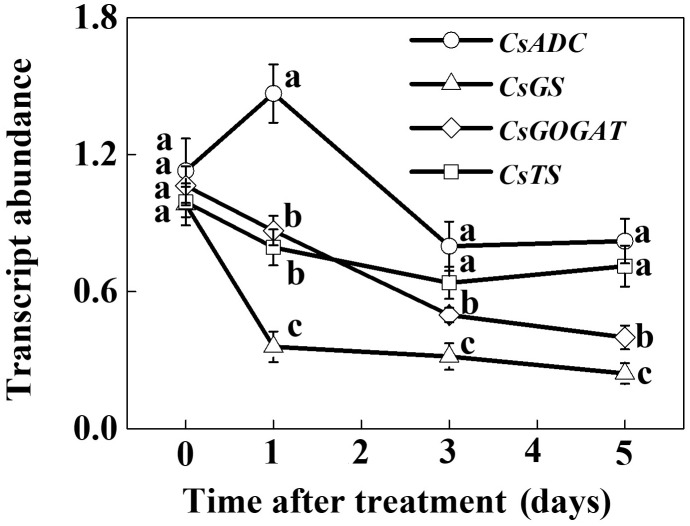
Time-course analysis of theanine biosynthetic genes as influenced by the SHT in tea leaves. Two years-old tea [*Camellia sinensis* (L.) O. Kuntze cv. Longjing 43] seedlings were exposed to SHT (35°C) for 5 days in temperature-controlled chambers. One bud and two leaves under the bud were sampled at the indicated time-points to analyze transcript abundance of *ARGININE DECARBOXYLASE* (*CsADC*), *GLUTAMINE SYNTHETASE* (*CsGS*), *GLUTAMATE SYNTHASE* (*CsGOGAT*), and *THEANINE SYNTHASE* (*CsTS*) using real-time qPCR. Data are presented as the mean of three biological replicates (±SD). Means denoted by the same letter at the same time point do not significantly differ at *P* < 0.05 according to Tukey’s test.

To assess the effect of SHT on theanine accumulation in tea leaves, we kept a group of tea plants at 35°C for 5 days in a controlled growth chamber. Time-course analysis of theanine concentration revealed that SHT gradually decreased theanine concentration over time (**Figure 2**). For example, theanine concentration decreased by 8.40, 19.52, 23.52, 31.54, and 29.99% following exposure of tea plants to SHT for 1, 2, 3, 4, and 5 days, respectively. To further understand, how SHT subdues theanine concentration in tea leaves, we performed a time-course analysis of genes that encode key enzymes involved in theanine biosynthesis, such as ADC, GS, GOGAT, and TS. The results showed that transcript abundance of *GS*, *GOGAT*, and *TS* consistently decreased soon after imposition of SHT, whereas *GS* exhibited maximum decreases as compared to the others (**Figure 3**). Notably, SHT decreased transcript levels of *GS* by 63.47, 67.84, and 75.39% after 1, 3, and 5 days heat stress, respectively. Next to *GS*, SHT decreased *GOGAT* expression by 18.49, 53.21, and 62.44%, respectively. Similarly, although at a lower magnitude, SHT decreased *TS* expression by 20.07, 35.70, and 28.31 after 1, 3, and 5 days heat stress, respectively. On the other hand, *ADC* expression, however, initially increased by 29.99 following 1d SHT treatment, and then sharply decreased upto 3 days, which then remained constant until 5 days. This implies that decreased theanine concentration following SHT is largely attributed to the reduced transcription of *GS* and *GOGAT* under SHT and that such significant reductions in theanine concentrations are closely associated with the SHT-induced inhibition in theanine biosynthesis.

### Brassinosteroid Improves Theanine Concentration in Tea Leaves Under SHT

**FIGURE 4:**
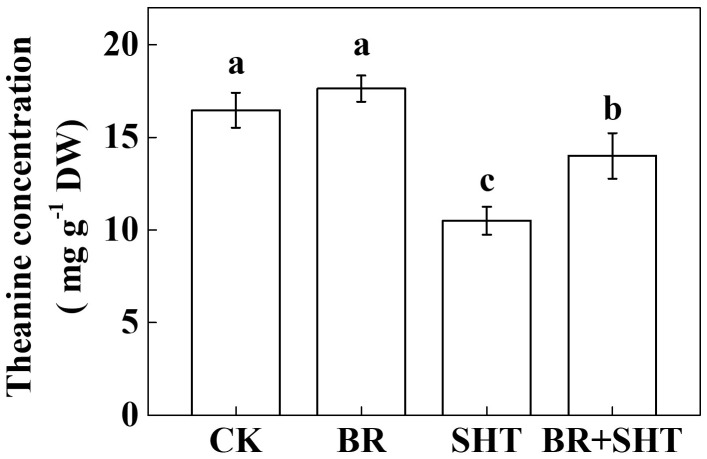
Effect of exogenous brassinosteroid on theanine concentration in tea leaves under normal and SHT conditions. CK, Control; BR, 24-epibrassinolide; SHT, sub high temperature. Foliar portion of 2 years-old tea [*C. sinensis* (L.) O. Kuntze cv. Longjing 43] seedlings were pretreated with 0.2 ppm BR until surface run-off. Twelve hours later, seedlings were exposed to SHT (35°C) for 5 days in temperature-controlled chambers. BR was reapplied 12 h prior to sample harvesting. Control plants were sprayed with ddH_2_O and kept under normal temperature (25°C). Data are presented as the mean of six replicates (±SD). Different letters indicate significant differences (*P* < 0.05) according to the Tukey’s test.

**FIGURE 5:**
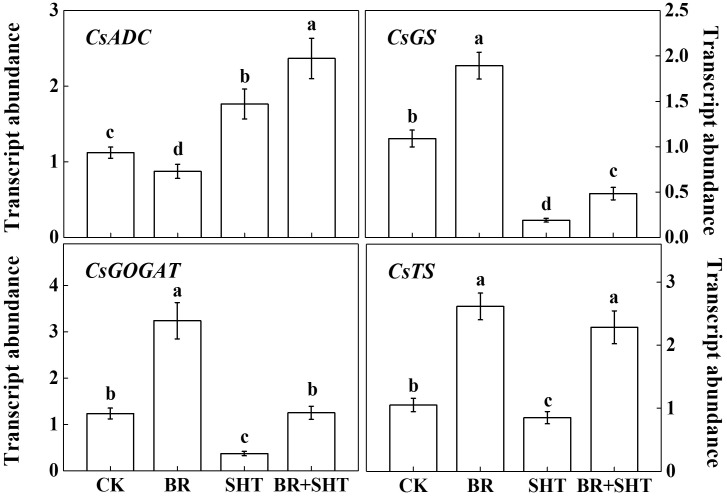
Effect of exogenous brassinosteroid and SHT on the transcripts of genes involved in theanine biosynthetic pathways. CK, Control; BR, 24-epibrassinolide; SHT, sub high temperature. Foliar portion of 2 years-old tea [*C. sinensis* (L.) O. Kuntze cv. Longjing 43] seedlings were pretreated with 0.2 ppm BR until surface run-off. Twelve hours later, seedlings were exposed to SHT (35°C) for 5 days in temperature-controlled chambers. BR was reapplied 12 h prior to sample harvesting. Control plants were sprayed with ddH_2_O and kept under normal temperature (25°C). One bud and two leaves under the bud were sampled after 5 days of the SHT treatment to analyze transcript abundance of *ARGININE DECARBOXYLASE* (*CsADC*), *GLUTAMINE SYNTHETASE* (*CsGS*), *GLUTAMATE SYNTHASE* (*CsGOGAT*), and *THEANINE SYNTHASE* (*CsTS*) using real-time qPCR. Data are presented as the mean of three biological replicates (±SD). Different letters indicate significant differences (*P* < 0.05) according to the Tukey’s test.

To examine whether BR can improve theanine concentration under SHT, we sprayed tea leaves with 0.2 ppm 24-epibrassinolide prior to SHT treatment. Although BR had no significant effect on the concentration of theanine under normal temperature, BR significantly increased theanine concentration under SHT (**Figure 4**). For instance, SHT decreased theanine concentration by 36.23% compared to the control; however, exogenous BR increased theanine concentration by 33.33% as compared to only SHT treatment. Next, we analyzed expression levels of key genes involved in theanine biosynthesis. Compared to control, BR only treatment upregulated expression of *GS*, *GOGAT* and *TS*, but downregulated that of *ADC* (**Figure 5**). When we compared expression of these genes between control and SHT, we found that SHT drastically inhibited *GS*, *GOGAT*, and *TS* expression by 82.57, 70.16, and 19.05%, respectively; however, *ADC* expression upregulated by 57.14% after SHT. Interestingly, BR application with SHT upregulated expression of all those genes over the only SHT treatment. For example, BR treatment with SHT upregulated expression levels of *ADC, GS, GOGAT*, and *TS* by 34.66, 152.63, 237.84, and 168.24%, respectively, when compared with SHT only treatment, indicating that BR-induced augmentation in theanine concentration under SHT is mainly contributed by the BR-induced enhancement in theanine biosynthesis.

### GS and GOGAT Play Critical Role in Sustaining Theanine Concentration in Tea Leaves Under SHT

**FIGURE 6:**
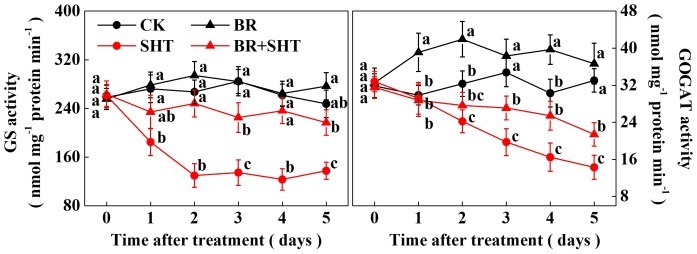
Time-course effect of SHT on the activity of key enzymes involved in theanine biosynthesis in tea leaves. Two years-old tea [*C. sinensis* (L.) O. Kuntze cv. Longjing 43] seedlings were exposed to SHT (SHT, 35°C) for 5 days in temperature-controlled chambers. Tea seedlings were pretreated with 0.2 ppm 24-epibrassinolide until surface run-off, 12 h prior to imposition of the SHT treatment. One bud and two leaves under the bud were sampled at the indicated time-points to analyze glutamine synthetase (GS) and glutamate synthase (GOGAT) activity. Data are presented as the mean of six replicates (±SD). Means denoted by the same letter at the same time point do not significantly differ at *P* < 0.05 according to Tukey’s test.

Since we noticed a high sensitivity of *GS* and *GOGAT* expression in response to SHT in tea leaves, we performed a time-course analysis of their activities following BR and/or SHT treatment (**Figure 6**). The results showed that GS activity drastically reduced by 32.14% after 1 day SHT treatment; while GOGAT decreased by only 2.26% compared to the control. Afterward GS activity decreased by 51.44, 52.71, 52.88, and 44.46% following 2, 3, 4, and 5 days SHT treatment. It appeared that decline in GS activity after 2 days of SHT treatment became stable. Although reduction in GOGAT activity was relatively low at 1 day SHT treatment, GOGAT activity gradually decreased over time accounting for 25.10, 43.20, 45.63, and 56.80% following 2, 3, 4, and 5 days SHT treatment, respectively. Thus, after 5 days SHT treatment, maximum reduction was noticed for GOGAT followed by GS when compared with the control. However, exogenous BR treatment maintained an increased GS activity following 2 days and onward SHT treatment in tea leaves as compared with SHT only treatment. Meanwhile, GOGAT activity in tea leaves under BR+SHT treatment was higher than that of SHT only treatment following 3, 4, and 5 days SHT treatment.

## Discussion

Temperature is an important environmental factor that impacts the yield and quality of many crops including tea plants (Ahmed et al., 2014; Ahammed et al., 2016; Li et al., 2016). Elevated leaf temperatures during the drought bring forth significant changes in the synthesis of tea phytochemicals (Jeyaramraja et al., 2003). When it comes to the theanine content in green tea, high temperature during post-harvest spreading exerts a stronger negative effect than low temperature, which eventually decreases theanine content by suppressing theanine biosynthetic gene expression (Liu et al., 2017a). Similar to the post-harvest effect of high temperature on theanine content, here *in planta* study reveals that a SHT could gradually decline theanine accumulation in tea leaves (**Figure 2**). The decline in theanine content was more or less attributed to the gradual downregulation of theanine biosynthetic genes, especially transcript level of *CsGOGAT*, which showed almost similar trend as of theanine concentration (**Figure 3**). On the contrary, exogenous BR significantly improved theanine concentration, which was attributed to remarkable upregulation in the transcript levels of theanine biosynthetic genes such as *CsGOGAT*, *CsGS*, *CsADC*, and *CsTS* (**Figures 4**, **5**). Time-course analysis of GOGAT and GS activity shows that exogenous BR helps tea plants to maintain an increased GOGAT and GS activity under SHT (**Figure 6**), which might greatly contribute to the enhanced theanine accumulation by BR pretreatment during SHT.

Theanine is an essential constituent of the green tea that imparts the so called ‘umami’ taste (Deng et al., 2010; Vuong et al., 2011). In the current study, SHT-induced decline in theanine content was sharp during initial 4 days after imposition of heat treatment (**Figure 2**). Afterward, no further decline in theanine content was noticed, indicating a potential acclimation response to the SHT. Theanine accumulation in tea leaves depends on its synthesis, transportation and hydrolysis (Deng et al., 2008, 2010; Liu et al., 2017b). Importantly, hydrolysis appears to be minor determinant under perturbed temperatures as SHTs suppress enzyme activity and genes involved in theanine biosynthesis (**Figures 5**, **6**). However, rapid loss of water due to high temperature-induced evaporation makes nitrogenous compounds (such as theanine) more concentrated and increases exposure to hydrolases. This phenomenon eventually enhances hydrolysis of theanine, leading to decreased theanine content in tea leaves (Liu et al., 2017a). In addition, high temperature can potentially hydrolyze the key enzymes, such as GOGAT and GS in theanine biosynthetic pathway, which drastically inhibits theanine biosynthesis.

Although a range of plant species possess the enzymes or genes that catalyze the conversion of ethylamine and L-glutamic acid to L-theanine (Mu et al., 2015), ethylamine explicitly accumulates in *Camellia* species, particularly in *C. sinensis*, suggesting that ethylamine availability is the key determinant for the elevated accumulation of L-theanine in tea leaves compared to other plants such as *Zea mays*, *Arabidopsis thaliana*, and *Solanum lycopersicum* (Cheng et al., 2017). *S*-adenosylmethionine decarboxylase (SAMDC) and arginine decarboxylase (ADC) are main enzymes in polyamine metabolism, however, they play similar roles with L-alanine decarboxylase (AIDA) that converts L-Alanine to ethylamine (Liu et al., 2017b). In the current study, SHT slightly upregulated transcript level of *ADC* gene; however, BR pretreatment with SHT caused a dramatic upregulation in *ADC* gene expression (**Figure 5**), indicating that BR improves theanine accumulation by enhancing metabolic flow of ethylamine through transcriptional regulation. Meanwhile, SHT downregulated transcript levels of *GS*, *GOGAT*, and *TS*, which was consistent with the effect of drought that declined theanine content by suppressing the expression levels of *GOGAT*, *GDH*, and *TS* (Wang et al., 2016). By contrast, exogenous BR pretreatment on SHT plants increased transcript levels of *GS*, *GOGAT*, and *TS* as compared to that in only SHT plants (**Figure 5**). Therefore, it is convincing that BR treatment can enhance *in vivo* accumulation of theanine in tea leaves by regulating transcripts of theanine biosynthetic genes. At this point, it is difficult to speculate how BR regulates theanine biosynthesis at transcriptional level. Previous studies have shown that signaling molecule nitric oxide is involved in BR-mediated flavonoid biosynthesis in tea plants (Li et al., 2017a). Therefore, it will be interesting to further explore the role of reactive oxygen species and reactive nitrogen species in BR-mediated theanine biosynthesis.

Glutamine synthetase/glutamate synthase is one of the key enzymes that plays critical role in theanine biosynthesis. It participates in the GS/GOGAT cycle, through which glutamate, the precursor for the biosynthesis of theanine is derived (Deng et al., 2008). To obtain a better insight into the SHT- and/or BR-induced changes in GS and GOGAT, we performed a time-course analysis of their activities (**Figure 6**). While GS activity was not much affected by BR in the absence of SHT, GOGAT activity was noticeable promoted by BR only treatment. More importantly, SHT-induced declines in GS and GOGAT activity were greatly attenuated by BR treatment, suggesting that BR-induced regulation of GOGAT activity plays essential role in maintaining theanine content in tea leaves under SHT, which is consistent with the central position of GOGATs in theanine biosynthetic pathway in tea plants (Liu et al., 2017a).

A number of studies show that the quality of tea fluctuates seasonally which is closely associated with the changes in the chemical composition (Xu et al., 2012; Dai et al., 2015; Liu J. et al., 2016). For instance, in summer tea, bitterness and astringency rise, whereas umami taste declines as compared to spring tea (Xu et al., 2012). Weather parameters such as temperature and sunshine remain elevated in the summer, which impact several flavonoids and amino acids responsible for the seasonal variations in tea quality (Dai et al., 2015). In this study, we explored profound effects of SHT and BR on theanine which influences the favorable taste of tea. Our findings indicate that BR promotes *in vivo* accumulation of theanine by selective regulation of transcript of theanine biosynthetic genes (**Figures 3**, **5**). In addition to the medicinal function and quality of tea, theanine plays important physiological roles in plants (Deng et al., 2012). As theanine comprises 50% of total amino acids in tea leaves, BR-induced enhanced theanine may also function as an energy source and as precursors for the biosynthesis of many bioactive molecules that eventually enhance tolerance to high temperature (Wu, 2009).

## Author Contributions

XL and W-YH conceived and designed the research. XL, J-PW, YL, and LZ performed the experiments and analyzed the data. W-YH provided crucial reagents and supervised the study. GA and XL wrote the manuscript. All authors reviewed the manuscript.

## Conflict of Interest Statement

The authors declare that the research was conducted in the absence of any commercial or financial relationships that could be construed as a potential conflict of interest.
