# Supplementary material for: Brassinosteroids Attenuate Moderate High Temperature-Caused Decline in Tea Quality by Enhancing Theanine Biosynthesis in Camellia sinensis L
Source: Front Plant Sci. 2018 Jul 24;9:1016. doi: 10.3389/fpls.2018.01016 (PMC6066615; doi:10.3389/fpls.2018.01016)
Supplement: Supplementary file 1 [file Table_1.DOCX]

**Supplemental Table S1.** Primers used for real time RT-PCR assays.

| **Gene** | **Functional annotation** | **Accession** | **Primer pairs** |
| --- | --- | --- | --- |
| *CsPTB* | *POLYPYRIMIDINERACR-BINDING PROTEIN* | GAAC01052498.1 | F: 5’- TGACCAAGCACACTCCACACTATCG-3’ |
|  |  |  | R: 5’- TGCCCCCTTATCATCATCCACAA-3’ |
| *CsADC* | *ARGININE DECARBOXYLASE* | JQ653274 | F: 5’- TCTCAGATACCCACAACCAC-3’ |
|  |  |  | R: 5’- GCCCATAACTAACCGAAATA-3’ |
| *CsGS* | [*GLUTAMINE SYNTHETASE*](http://www.ncbi.nlm.nih.gov/protein/NP_001234126.1) | EU284131 | F: 5’- GGAGGTTATCCTGGACCTCA-3’ |
|  |  |  | R: 5’- GGCAAGCCTTGTAGTGTGAA-3’ |
| *CsGOGAT* | *GLUTAMINE: 2-OXOGLUTARATE* | JN602371 | F: 5’-ACACTGCCACATCTCAAAGG-3’ |
|  | *AMINOTRANSFERASE* |  | R: 5’-CCAATTGATCAGCATTGACC-3’ |
| *CsTS* | *THEANINE SYNTHASE* | DD401895 | F: 5’-TCTTTCTGGACCTGTGAGTG-3’ |
|  |  |  | R: 5’-GCTTGAGGGTAGATAATGAGT-3’ |
